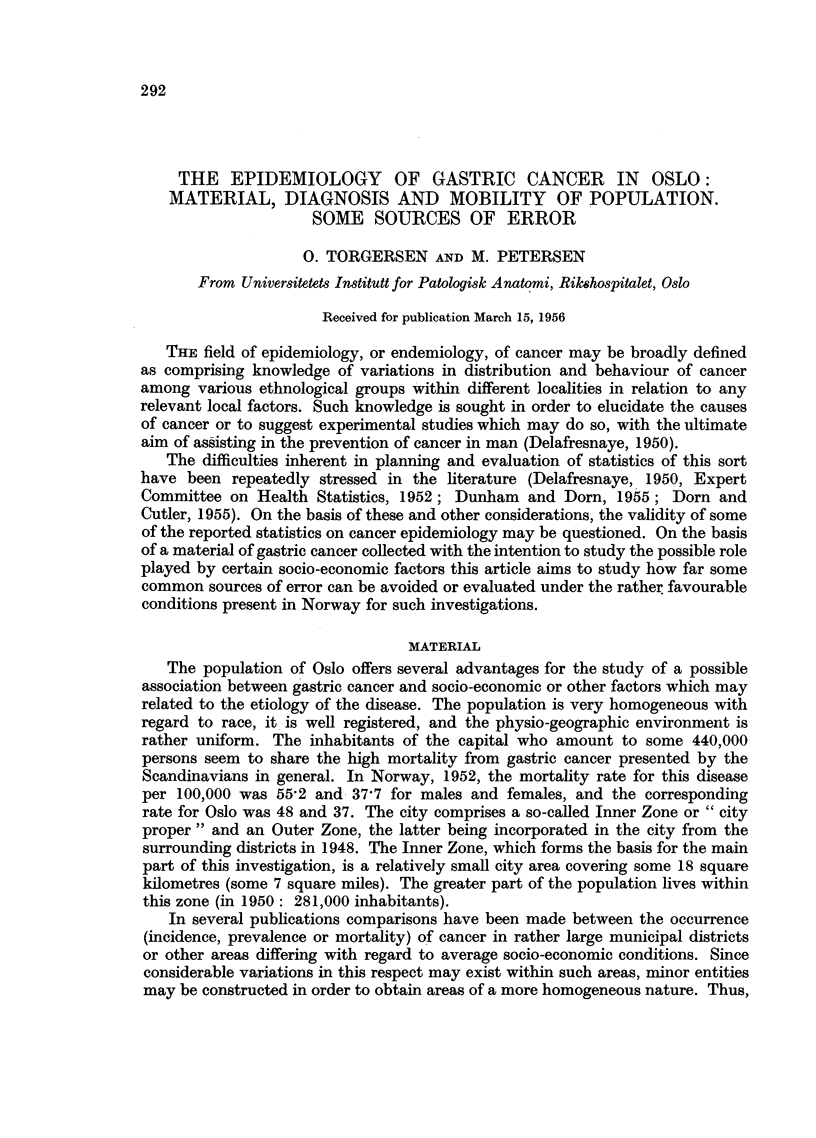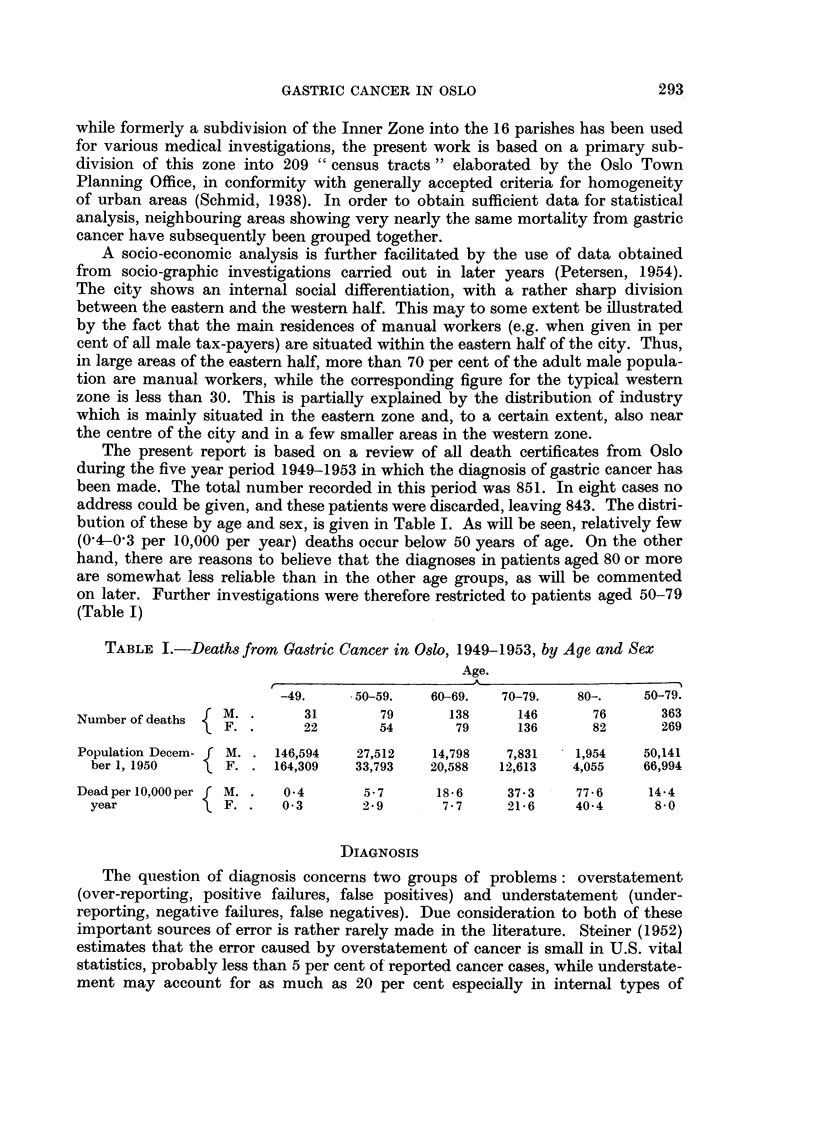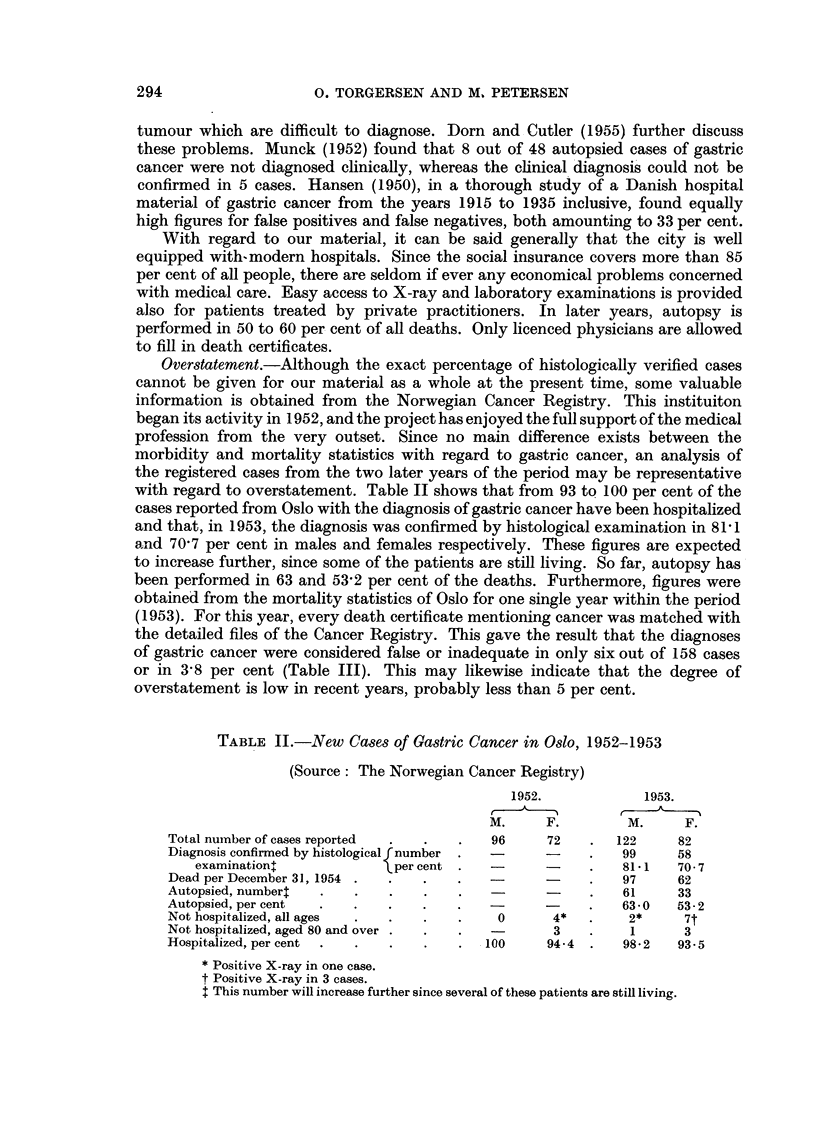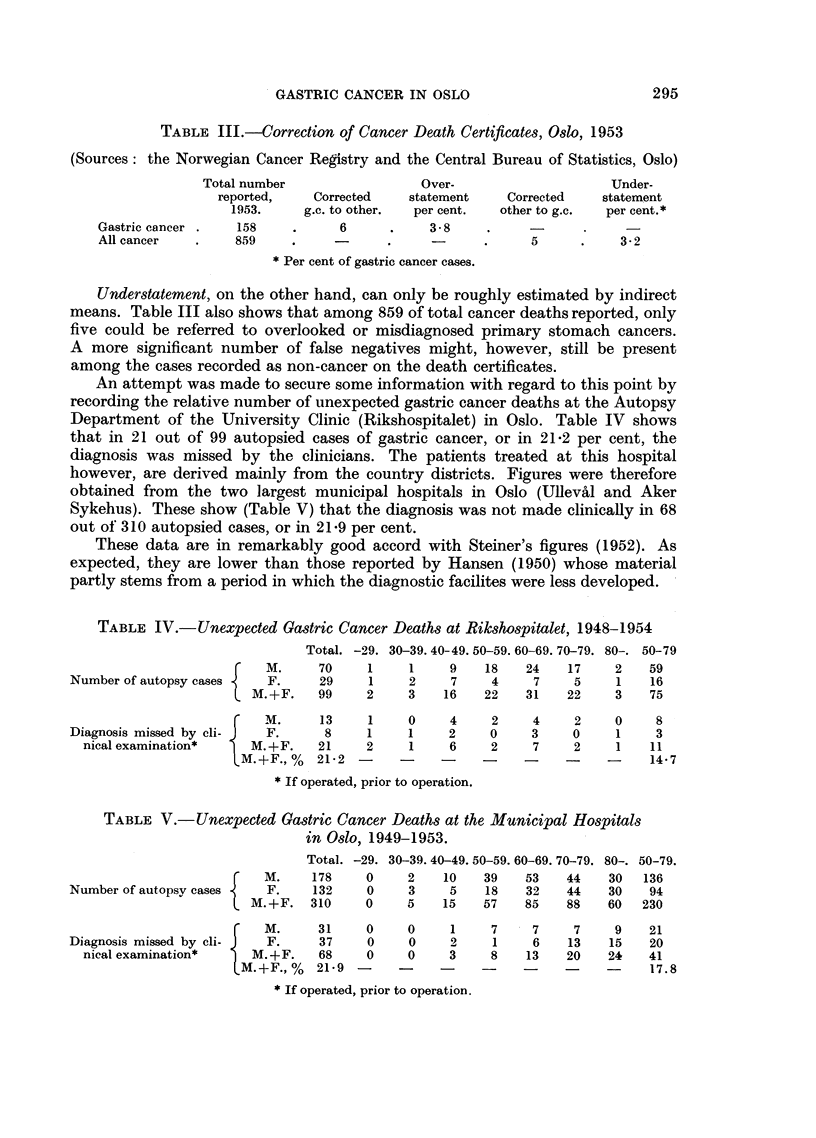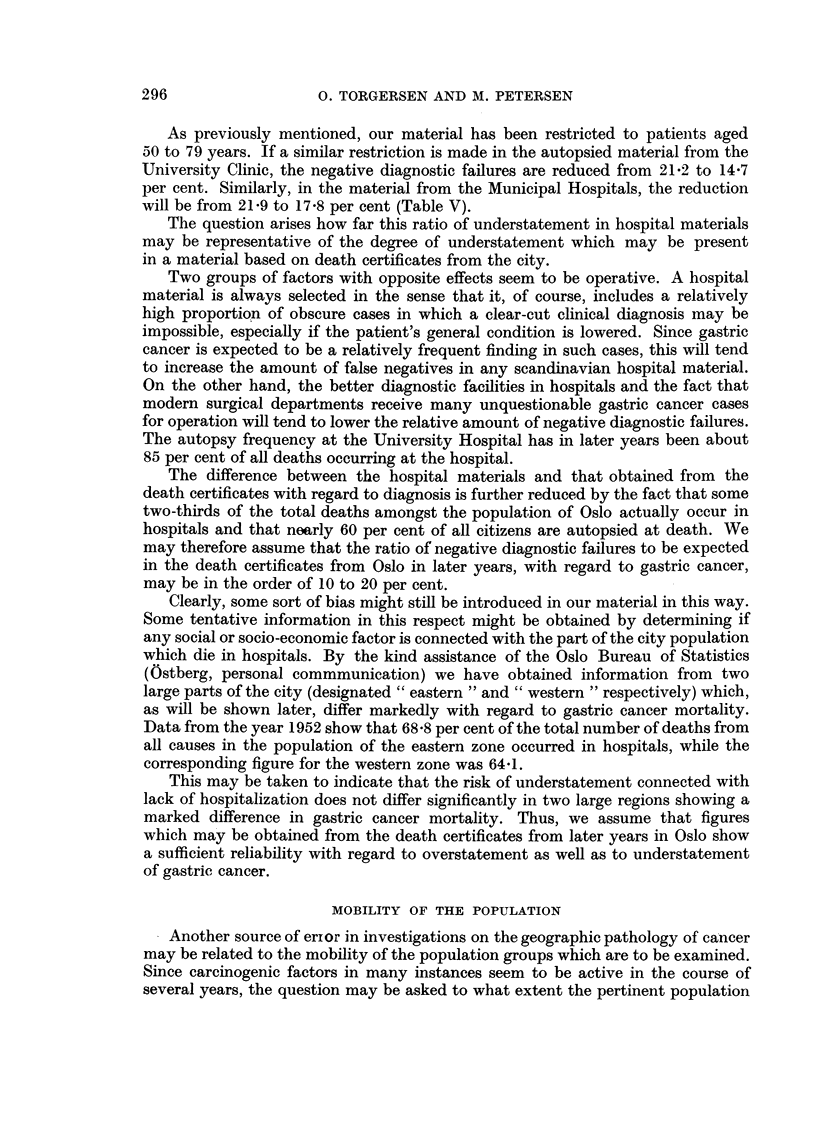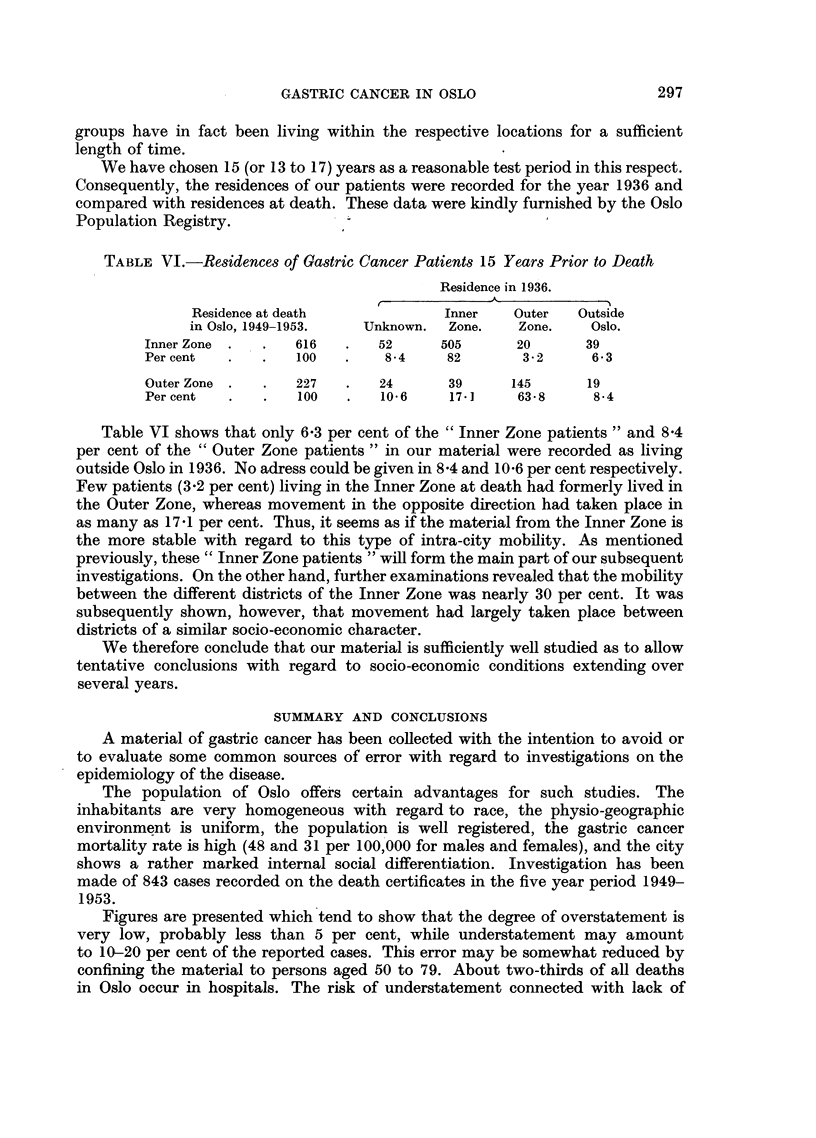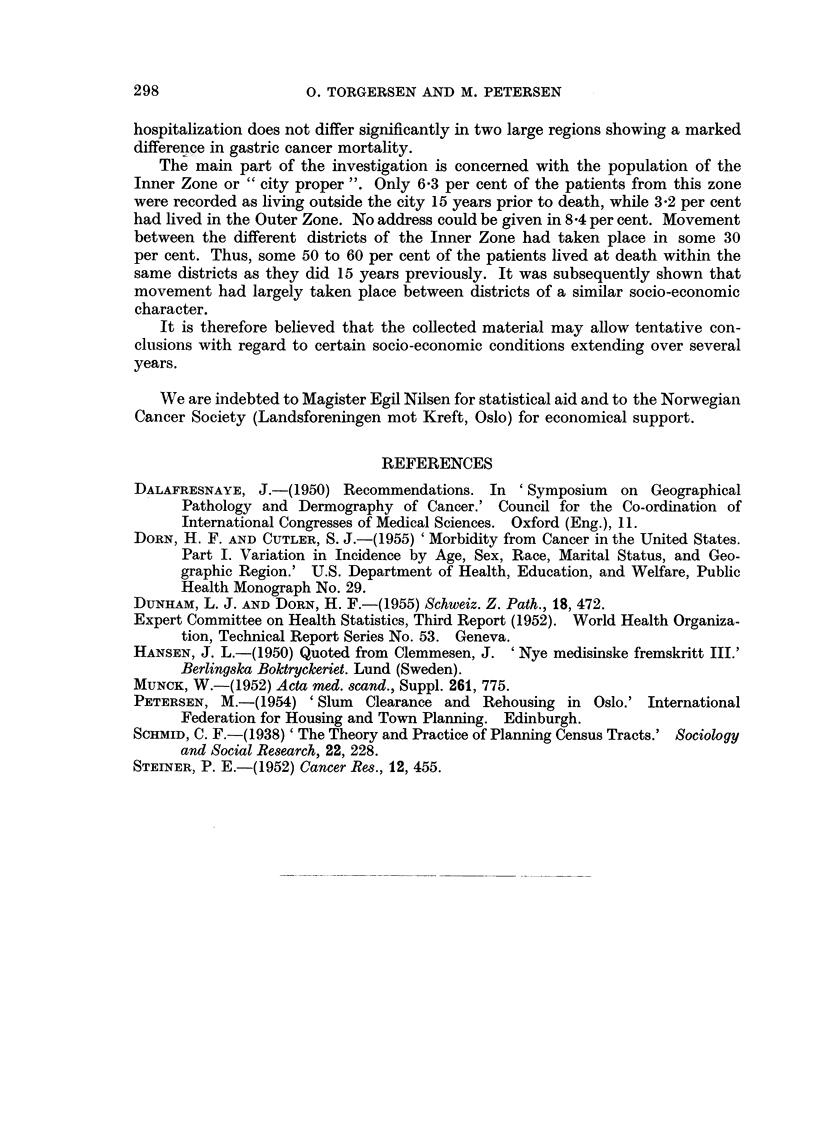# The Epidemiology of Gastric Cancer in Oslo: Material, Diagnosis and Mobility of Population. Some Sources of Error

**DOI:** 10.1038/bjc.1956.33

**Published:** 1956-06

**Authors:** O. Torgersen, M. Petersen


### THE EPIDEMIOLOGY OF GASTRIC CANCER IN OSLO: MATERIAL, DIAGNOSIS AND MOBILITY OF POPULATION. SOME SOURCES OF ERROR

O. TORGERSEN AND M. PETERSEN

*From Universitetets Institutt for Patologisk Anatomi, Rikshospitalet, Oslo*

Received for publication March 15, 1956

THE field of epidemiology, or endemiology, of cancer may be broadly defined as comprising knowledge of variations in distribution and behaviour of cancer among various ethnological groups within different localities in relation to any relevant local factors. Such knowledge is sought in order to elucidate the causes of cancer or to suggest experimental studies which may do so, with the ultimate aim of assisting in the prevention of cancer in man (Delafresnaye, 1950).

The difficulties inherent in planning and evaluation of statistics of this sort have been repeatedly stressed in the literature (Delafresnaye, 1950, Expert Committee on Health Statistics, 1952; Dunham and Dorn, 1955; Dorn and Cutler, 1955). On the basis of these and other considerations, the validity of some of the reported statistics on cancer epidemiology may be questioned. On the basis of a material of gastric cancer collected with the intention to study the possible role played by certain socio-economic factors this article aims to study how far some common sources of error can be avoided or evaluated under the rather favourable conditions present in Norway for such investigations.

### MATERIAL

The population of Oslo offers several advantages for the study of a possible association between gastric cancer and socio-economic or other factors which may related to the etiology of the disease. The population is very homogeneous with regard to race, it is well registered, and the physio-geographic environment is rather uniform. The inhabitants of the capital who amount to some 440,000 persons seem to share the high mortality from gastric cancer presented by the Scandinavians in general. In Norway, 1952, the mortality rate for this disease per 100,000 was 55·2 and 37·7 for males and females, and the corresponding rate for Oslo was 48 and 37. The city comprises a so-called Inner Zone or "city proper" and an Outer Zone, the latter being incorporated in the city from the surrounding districts in 1948. The Inner Zone, which forms the basis for the main part of this investigation, is a relatively small city area covering some 18 square kilometres (some 7 square miles). The greater part of the population lives within this zone (in 1950: 281,000 inhabitants).

In several publications comparisons have been made between the occurrence (incidence, prevalence or mortality) of cancer in rather large municipal districts or other areas differing with regard to average socio-economic conditions. Since considerable variations in this respect may exist within such areas, minor entities may be constructed in order to obtain areas of a more homogeneous nature. Thus, while formerly a subdivision of the Inner Zone into the 16 parishes has been used for various medical investigations, the present work is based on a primary subdivision of this zone into 209 " census tracts " elaborated by the Oslo Town Planning Office, in conformity with generally accepted criteria for homogeneity of urban areas (Schmid, 1938). In order to obtain sufficient data for statistical analysis, neighbouring areas showing very nearly the same mortality from gastric cancer have subsequently been grouped together.

A socio-economic analysis is further facilitated by the use of data obtained from socio-graphic investigations carried out in later years (Petersen, 1954). The city shows an internal social differentiation, with a rather sharp division between the eastern and the western half. This may to some extent be illustrated by the fact that the main residences of manual workers (e.g. when given in per cent of all male tax-payers) are situated within the eastern half of the city. Thus, in large areas of the eastern half, more than 70 per cent of the adult male population are manual workers, while the corresponding figure for the typical western zone is less than 30. This is partially explained by the distribution of industry which is mainly situated in the eastern zone and, to a certain extent, also near the centre of the city and in a few smaller areas in the western zone.

The present report is based on a review of all death certificates from Oslo during the five year period 1949–1953 in which the diagnosis of gastric cancer has been made. The total number recorded in this period was 851. In eight cases no address could be given, and these patients were discarded, leaving 843. The distribution of these by age and sex, is given in Table I. As will be seen, relatively few (0·4–0·3 per 10,000 per year) deaths occur below 50 years of age. On the other hand, there are reasons to believe that the diagnoses in patients aged 80 or more are somewhat less reliable than in the other age groups, as will be commented on later. Further investigations were therefore restricted to patients aged 50–79 (Table I)

TABLE I.—*Deaths from Gastric Cancer in Oslo*, 1949–1953, *by Age and Sex*

| | | Age. | | | | | |
|---|---|---|---|---|---|---|---|
| | | –49. | 50–59. | 60–69. | 70–79. | 80–. | 50–79. |
| Number of deaths | M. | 31 | 79 | 138 | 146 | 76 | 363 |
| | F. | 22 | 54 | 79 | 136 | 82 | 269 |
| Population December 1, 1950 | M. | 146,594 | 27,512 | 14,798 | 7,831 | 1,954 | 50,141 |
| | F. | 164,309 | 33,793 | 20,588 | 12,613 | 4,055 | 66,994 |
| Dead per 10,000 per year | M. | 0·4 | 5·7 | 18·6 | 37·3 | 77·6 | 14·4 |
| | F. | 0·3 | 2·9 | 7·7 | 21·6 | 40·4 | 8·0 |

### DIAGNOSIS

The question of diagnosis concerns two groups of problems: overstatement (over-reporting, positive failures, false positives) and understatement (under-reporting, negative failures, false negatives). Due consideration to both of these important sources of error is rather rarely made in the literature. Steiner (1952) estimates that the error caused by overstatement of cancer is small in U.S. vital statistics, probably less than 5 per cent of reported cancer cases, while understatement may account for as much as 20 per cent especially in internal types of tumour which are difficult to diagnose. Dorn and Cutler (1955) further discuss these problems. Munck (1952) found that 8 out of 48 autopsied cases of gastric cancer were not diagnosed clinically, whereas the clinical diagnosis could not be confirmed in 5 cases. Hansen (1950), in a thorough study of a Danish hospital material of gastric cancer from the years 1915 to 1935 inclusive, found equally high figures for false positives and false negatives, both amounting to 33 per cent.

With regard to our material, it can be said generally that the city is well equipped with modern hospitals. Since the social insurance covers more than 85 per cent of all people, there are seldom if ever any economical problems concerned with medical care. Easy access to X-ray and laboratory examinations is provided also for patients treated by private practitioners. In later years, autopsy is performed in 50 to 60 per cent of all deaths. Only licenced physicians are allowed to fill in death certificates.

*Overstatement.*—Although the exact percentage of histologically verified cases cannot be given for our material as a whole at the present time, some valuable information is obtained from the Norwegian Cancer Registry. This instituiton began its activity in 1952, and the project has enjoyed the full support of the medical profession from the very outset. Since no main difference exists between the morbidity and mortality statistics with regard to gastric cancer, an analysis of the registered cases from the two later years of the period may be representative with regard to overstatement. Table II shows that from 93 to 100 per cent of the cases reported from Oslo with the diagnosis of gastric cancer have been hospitalized and that, in 1953, the diagnosis was confirmed by histological examination in 81·1 and 70·7 per cent in males and females respectively. These figures are expected to increase further, since some of the patients are still living. So far, autopsy has been performed in 63 and 53·2 per cent of the deaths. Furthermore, figures were obtained from the mortality statistics of Oslo for one single year within the period (1953). For this year, every death certificate mentioning cancer was matched with the detailed files of the Cancer Registry. This gave the result that the diagnoses of gastric cancer were considered false or inadequate in only six out of 158 cases or in 3·8 per cent (Table III). This may likewise indicate that the degree of overstatement is low in recent years, probably less than 5 per cent.

TABLE II.—*New Cases of Gastric Cancer in Oslo*, 1952–1953

(Source : The Norwegian Cancer Registry)

| | 1952. | | 1953. | |
|---|---|---|---|---|
| | M. | F. | M. | F. |
| Total number of cases reported | 96 | 72 | 122 | 82 |
| Diagnosis confirmed by histological examination‡ — number | — | — | 99 | 58 |
| Diagnosis confirmed by histological examination‡ — per cent | — | — | 81·1 | 70·7 |
| Dead per December 31, 1954 | — | — | 97 | 62 |
| Autopsied, number‡ | — | — | 61 | 33 |
| Autopsied, per cent | — | — | 63·0 | 53·2 |
| Not hospitalized, all ages | 0 | 4* | 2* | 7† |
| Not hospitalized, aged 80 and over | — | 3 | 1 | 3 |
| Hospitalized, per cent | 100 | 94·4 | 98·2 | 93·5 |

* Positive X-ray in one case.
† Positive X-ray in 3 cases.
‡ This number will increase further since several of these patients are still living.

TABLE III.—*Correction of Cancer Death Certificates, Oslo,* 1953

(Sources: the Norwegian Cancer Registry and the Central Bureau of Statistics, Oslo)

| | Total number reported, 1953. | Corrected g.c. to other. | Over-statement per cent. | Corrected other to g.c. | Under-statement per cent.* |
|---|---|---|---|---|---|
| Gastric cancer | 158 | 6 | 3·8 | — | — |
| All cancer | 859 | — | — | 5 | 3·2 |

* Per cent of gastric cancer cases.

*Understatement*, on the other hand, can only be roughly estimated by indirect means. Table III also shows that among 859 of total cancer deaths reported, only five could be referred to overlooked or misdiagnosed primary stomach cancers. A more significant number of false negatives might, however, still be present among the cases recorded as non-cancer on the death certificates.

An attempt was made to secure some information with regard to this point by recording the relative number of unexpected gastric cancer deaths at the Autopsy Department of the University Clinic (Rikshospitalet) in Oslo. Table IV shows that in 21 out of 99 autopsied cases of gastric cancer, or in 21·2 per cent, the diagnosis was missed by the clinicians. The patients treated at this hospital however, are derived mainly from the country districts. Figures were therefore obtained from the two largest municipal hospitals in Oslo (Ullevål and Aker Sykehus). These show (Table V) that the diagnosis was not made clinically in 68 out of 310 autopsied cases, or in 21·9 per cent.

These data are in remarkably good accord with Steiner's figures (1952). As expected, they are lower than those reported by Hansen (1950) whose material partly stems from a period in which the diagnostic facilites were less developed.

TABLE IV.—*Unexpected Gastric Cancer Deaths at Rikshospitalet,* 1948–1954

| | | Total. | -29. | 30–39. | 40–49. | 50–59. | 60–69. | 70–79. | 80–. | 50–79 |
|---|---|---|---|---|---|---|---|---|---|---|
| Number of autopsy cases | M. | 70 | 1 | 1 | 9 | 18 | 24 | 17 | 2 | 59 |
| | F. | 29 | 1 | 2 | 7 | 4 | 7 | 5 | 1 | 16 |
| | M.+F. | 99 | 2 | 3 | 16 | 22 | 31 | 22 | 3 | 75 |
| Diagnosis missed by clinical examination* | M. | 13 | 1 | 0 | 4 | 2 | 4 | 2 | 0 | 8 |
| | F. | 8 | 1 | 1 | 2 | 0 | 3 | 0 | 1 | 3 |
| | M.+F. | 21 | 2 | 1 | 6 | 2 | 7 | 2 | 1 | 11 |
| | M.+F., % | 21·2 | — | — | — | — | — | — | — | 14·7 |

* If operated, prior to operation.

TABLE V.—*Unexpected Gastric Cancer Deaths at the Municipal Hospitals in Oslo,* 1949–1953.

| | | Total. | -29. | 30–39. | 40–49. | 50–59. | 60–69. | 70–79. | 80–. | 50–79. |
|---|---|---|---|---|---|---|---|---|---|---|
| Number of autopsy cases | M. | 178 | 0 | 2 | 10 | 39 | 53 | 44 | 30 | 136 |
| | F. | 132 | 0 | 3 | 5 | 18 | 32 | 44 | 30 | 94 |
| | M.+F. | 310 | 0 | 5 | 15 | 57 | 85 | 88 | 60 | 230 |
| Diagnosis missed by clinical examination* | M. | 31 | 0 | 0 | 1 | 7 | 7 | 7 | 9 | 21 |
| | F. | 37 | 0 | 0 | 2 | 1 | 6 | 13 | 15 | 20 |
| | M.+F. | 68 | 0 | 0 | 3 | 8 | 13 | 20 | 24 | 41 |
| | M.+F., % | 21·9 | — | — | — | — | — | — | — | 17.8 |

* If operated, prior to operation.

As previously mentioned, our material has been restricted to patients aged 50 to 79 years. If a similar restriction is made in the autopsied material from the University Clinic, the negative diagnostic failures are reduced from 21·2 to 14·7 per cent. Similarly, in the material from the Municipal Hospitals, the reduction will be from 21·9 to 17·8 per cent (Table V).

The question arises how far this ratio of understatement in hospital materials may be representative of the degree of understatement which may be present in a material based on death certificates from the city.

Two groups of factors with opposite effects seem to be operative. A hospital material is always selected in the sense that it, of course, includes a relatively high proportion of obscure cases in which a clear-cut clinical diagnosis may be impossible, especially if the patient's general condition is lowered. Since gastric cancer is expected to be a relatively frequent finding in such cases, this will tend to increase the amount of false negatives in any scandinavian hospital material. On the other hand, the better diagnostic facilities in hospitals and the fact that modern surgical departments receive many unquestionable gastric cancer cases for operation will tend to lower the relative amount of negative diagnostic failures. The autopsy frequency at the University Hospital has in later years been about 85 per cent of all deaths occurring at the hospital.

The difference between the hospital materials and that obtained from the death certificates with regard to diagnosis is further reduced by the fact that some two-thirds of the total deaths amongst the population of Oslo actually occur in hospitals and that nearly 60 per cent of all citizens are autopsied at death. We may therefore assume that the ratio of negative diagnostic failures to be expected in the death certificates from Oslo in later years, with regard to gastric cancer, may be in the order of 10 to 20 per cent.

Clearly, some sort of bias might still be introduced in our material in this way. Some tentative information in this respect might be obtained by determining if any social or socio-economic factor is connected with the part of the city population which die in hospitals. By the kind assistance of the Oslo Bureau of Statistics (Östberg, personal commmunication) we have obtained information from two large parts of the city (designated " eastern " and " western " respectively) which, as will be shown later, differ markedly with regard to gastric cancer mortality. Data from the year 1952 show that 68·8 per cent of the total number of deaths from all causes in the population of the eastern zone occurred in hospitals, while the corresponding figure for the western zone was 64·1.

This may be taken to indicate that the risk of understatement connected with lack of hospitalization does not differ significantly in two large regions showing a marked difference in gastric cancer mortality. Thus, we assume that figures which may be obtained from the death certificates from later years in Oslo show a sufficient reliability with regard to overstatement as well as to understatement of gastric cancer.

### MOBILITY OF THE POPULATION

Another source of error in investigations on the geographic pathology of cancer may be related to the mobility of the population groups which are to be examined. Since carcinogenic factors in many instances seem to be active in the course of several years, the question may be asked to what extent the pertinent population groups have in fact been living within the respective locations for a sufficient length of time.

We have chosen 15 (or 13 to 17) years as a reasonable test period in this respect. Consequently, the residences of our patients were recorded for the year 1936 and compared with residences at death. These data were kindly furnished by the Oslo Population Registry.

TABLE VI.—*Residences of Gastric Cancer Patients* 15 *Years Prior to Death*

| Residence at death in Oslo, 1949–1953. | | Residence in 1936. Unknown. | Inner Zone. | Outer Zone. | Outside Oslo. |
|---|---|---|---|---|---|
| Inner Zone | 616 | 52 | 505 | 20 | 39 |
| Per cent | 100 | 8·4 | 82 | 3·2 | 6·3 |
| Outer Zone | 227 | 24 | 39 | 145 | 19 |
| Per cent | 100 | 10·6 | 17·1 | 63·8 | 8·4 |

Table VI shows that only 6·3 per cent of the " Inner Zone patients " and 8·4 per cent of the " Outer Zone patients " in our material were recorded as living outside Oslo in 1936. No adress could be given in 8·4 and 10·6 per cent respectively. Few patients (3·2 per cent) living in the Inner Zone at death had formerly lived in the Outer Zone, whereas movement in the opposite direction had taken place in as many as 17·1 per cent. Thus, it seems as if the material from the Inner Zone is the more stable with regard to this type of intra-city mobility. As mentioned previously, these " Inner Zone patients " will form the main part of our subsequent investigations. On the other hand, further examinations revealed that the mobility between the different districts of the Inner Zone was nearly 30 per cent. It was subsequently shown, however, that movement had largely taken place between districts of a similar socio-economic character.

We therefore conclude that our material is sufficiently well studied as to allow tentative conclusions with regard to socio-economic conditions extending over several years.

### SUMMARY AND CONCLUSIONS

A material of gastric cancer has been collected with the intention to avoid or to evaluate some common sources of error with regard to investigations on the epidemiology of the disease.

The population of Oslo offers certain advantages for such studies. The inhabitants are very homogeneous with regard to race, the physio-geographic environment is uniform, the population is well registered, the gastric cancer mortality rate is high (48 and 31 per 100,000 for males and females), and the city shows a rather marked internal social differentiation. Investigation has been made of 843 cases recorded on the death certificates in the five year period 1949–1953.

Figures are presented which tend to show that the degree of overstatement is very low, probably less than 5 per cent, while understatement may amount to 10–20 per cent of the reported cases. This error may be somewhat reduced by confining the material to persons aged 50 to 79. About two-thirds of all deaths in Oslo occur in hospitals. The risk of understatement connected with lack of hospitalization does not differ significantly in two large regions showing a marked difference in gastric cancer mortality.

The main part of the investigation is concerned with the population of the Inner Zone or " city proper ". Only 6·3 per cent of the patients from this zone were recorded as living outside the city 15 years prior to death, while 3·2 per cent had lived in the Outer Zone. No address could be given in 8·4 per cent. Movement between the different districts of the Inner Zone had taken place in some 30 per cent. Thus, some 50 to 60 per cent of the patients lived at death within the same districts as they did 15 years previously. It was subsequently shown that movement had largely taken place between districts of a similar socio-economic character.

It is therefore believed that the collected material may allow tentative conclusions with regard to certain socio-economic conditions extending over several years.

We are indebted to Magister Egil Nilsen for statistical aid and to the Norwegian Cancer Society (Landsforeningen mot Kreft, Oslo) for economical support.

### REFERENCES

DALAFRESNAYE, J.—(1950) Recommendations. In 'Symposium on Geographical Pathology and Dermography of Cancer.' Council for the Co-ordination of International Congresses of Medical Sciences. Oxford (Eng.), 11.

DORN, H. F. AND CUTLER, S. J.—(1955) 'Morbidity from Cancer in the United States. Part I. Variation in Incidence by Age, Sex, Race, Marital Status, and Geographic Region.' U.S. Department of Health, Education, and Welfare, Public Health Monograph No. 29.

DUNHAM, L. J. AND DORN, H. F.—(1955) *Schweiz. Z. Path.*, **18**, 472.

Expert Committee on Health Statistics, Third Report (1952). World Health Organization, Technical Report Series No. 53. Geneva.

HANSEN, J. L.—(1950) Quoted from Clemmesen, J. 'Nye medisinske fremskritt III.' *Berlingska Boktryckeriet.* Lund (Sweden).

MUNCK, W.—(1952) *Acta med. scand.*, Suppl. **261**, 775.

PETERSEN, M.—(1954) 'Slum Clearance and Rehousing in Oslo.' International Federation for Housing and Town Planning. Edinburgh.

SCHMID, C. F.—(1938) 'The Theory and Practice of Planning Census Tracts.' *Sociology and Social Research*, **22**, 228.

STEINER, P. E.—(1952) *Cancer Res.*, **12**, 455.